# Finding Quasi-Optimal Network Topologies for Information Transmission in Active Networks

**DOI:** 10.1371/journal.pone.0003479

**Published:** 2008-10-22

**Authors:** Murilo S. Baptista, Josué X. de Carvalho, Mahir S. Hussein

**Affiliations:** 1 Max-Planck-Institut für Physik komplexer Systeme, Dresden, Deutschland; 2 Centro de Matemática da Universidade do Porto, Porto, Portugal; 3 Institute of Physics, University of São Paulo, São Paulo, Brasil; University of Glasgow, United Kingdom

## Abstract

This work clarifies the relation between network circuit (topology) and behaviour (information transmission and synchronization) in active networks, e.g. neural networks. As an application, we show how one can find network topologies that are able to transmit a large amount of information, possess a large number of communication channels, and are robust under large variations of the network coupling configuration. This theoretical approach is general and does not depend on the particular dynamic of the elements forming the network, since the network topology can be determined by finding a Laplacian matrix (the matrix that describes the connections and the coupling strengths among the elements) whose eigenvalues satisfy some special conditions. To illustrate our ideas and theoretical approaches, we use neural networks of electrically connected chaotic Hindmarsh-Rose neurons.

## Introduction

Given an arbitrary time dependent stimulus that externally excites an active network formed by systems that have some intrinsic dynamics (e.g. neurons and oscillators), how much information from such stimulus can be realized by measuring the time evolution of one of the elements of the network ? Determining how and how much information flows along anatomical brain paths is an important requirement for the understanding of how animals perceive their environment, learn and behave [1], [2], [3].

Even though the approaches of Ref. [1], [2], [3], [4], [5], [6] have brought considerable understanding on how and how much information from a stimulus is transmitted in a neural network, the relation between network circuits (topology) and information transmission in a neural as well as an active network is still awaiting a more quantitative description [7]. And that is the main thrust of the present manuscript, namely, to present a quantitative way to relate network topology with information in active networks. Since information might not always be easy to be measured or quantified in experiments, we endeavour to clarify the relation between information and synchronization, a phenomenon which is often not only possible to observe but also relatively easy to characterize.

We initially proceed along the same line as in Refs. [8], [9], and study the information transfer in autonomous systems. However, instead of treating the information transfer between dynamical systems components, we treat the transfer of information per unit time exchanged between two elements in an autonomous chaotic active network. Thus, we neglect the complex relation between external stimulus and the network and show how to calculate an upper bound value for the mutual information rate (MIR) exchanged between two elements (a communication channel) in an autonomous network. Ultimately, we discuss how to extend this formula to non-chaotic networks suffering the influence of a time-dependent stimulus.

Most of this work is directed to ensure the plausibility and validity of the proposed formula for the upper bound of MIR (Sec. Results) and also to study its applications in order to clarify the relation among network topology, information, and synchronization. We do not rely only on results provided by this formula, but we also calculate the MIR by the methods in Refs. [10], [11] and by symbolic encoding the trajectory of the elements forming the network and then measuring the mutual information provided by this discrete sequence of symbols.

To illustrate the power of the proposed formula, we applied it to study the exchange of information in networks of coupled chaotic maps (Sec. Methods) and in Hindmarsh-Rose neural networks bidirectionally electrically coupled (Sec. Results). Our formula can be used to a larger class of active networks than the ones here considered. As the networks formed by elements coupled both electrically and chemically (see Ref. [12]). Still, the studied network topologies are much simpler than the ones found in the brain [13], [14]. Nevertheless, we do believe our approaches can be used to better understand how information is transfered in more realistic networks as the scale-free networks [15], the small-world networks [16], or power-law networks [17].

The analyses are carried out using quantities that we believe to be relevant to the treatment of information transmission in active networks: a *communication channel*, the *channel capacity*, and the *network capacity* (see definitions in Sec. Methods).

A communication channel represents a pathway through which information is exchanged. In this work, a communication channel is considered to be formed by a pair of elements. One element represents a transmitter and the other a receiver, where the information about the transmitter can be measured.

The channel capacity is defined in terms of the proposed upper bound for the MIR. It measures the local maximal rate of information that two elements in a given network are able to exchange, a point-to-point measure of information exchange. As we shall see, there are two network configurations for which the value of the upper bound can be considered to be maximal with respect to the coupling strength.

The network capacity is the maximum of the KS-entropy, for many possible network configurations with a given number of elements. It gives the amount of independent information that can be simultaneously transmitted within the whole network, and naturally bounds the value of the MIR in the channels, which concerns only the transmission of information between two elements.

While the channel capacity is bounded and does not depend on the number of elements forming the network, the network capacity depends on the number of elements forming the network.

As a direct application of the formula for the upper bound value of the MIR, we show that an active network can operate with a large amount of MIR and KS-entropy and at the same time it is robustly resistant to alterations in the coupling strengths, if the eigenvalues of the Laplacian matrix satisfy some specified conditions (Sec. Results). The Laplacian matrix describes the connections among the elements of the network.

The conditions on the eigenvalues depend on whether the network is constructed in order to possess communication channels that are either self-excitable or non-self-excitable (see definition in Sec. Methods). Active networks that possess non-self-excitable channels (formed by oscillators as the Rössler, or the Chua's circuit) have channels that achieve their capacity whenever their elements are in complete synchrony. Therefore, if a large amount of information is desired to be transmitted point-to-point in a non-self-excitable network, easily synchronizable networks are required. On the other hand, networks that possess self-excitable channels (as the ones formed by neurons), achieve simultaneously its channel and network capacities when there is at least one unstable mode of oscillation (time-scale) that is out of synchrony.

While non-self-excitable channels permit the exchanging of a moderate amount of information in a reliable fashion, due to the low level of desynchronization in the channel, self-excitable channels permit the exchange of surprisingly large amounts of information, not necessary reliable, due to the higher level of desynchronization in the channel.

We do not intend to find the best network topology among all possible ones. But rather, we aim at finding classes of network topologies that can not only transmit large amounts of information but are also robust under alterations in the coupling strengths. We arrive at two relevant eigenvalues conditions which provide networks that satisfy all these requirements. Either the network has elements that remain completely desynchronous for large variations of the coupling strength, forming the self-excitable channels, or the network has elements almost completely synchronous, forming the non-self-excitable channels. In fact, the studied network, a network formed by electrically connected Hindmarsh-Rose neurons [18], can have simultaneously self-excitable and non-self-excitable channels.

Self-excitable networks, namely those that have a majority number of self-excitable channels, have the topology of a perturbed star, i.e., they are composed of a central neuron connected to most of the other outer neurons, and some outer neurons sparsely connected among themselves. The networks that have non-self-excitable channels have the topology of a perturbed fully connected network, i.e., a network whose elements are almost all-to-all connected. The self-excitable network has thus a topology which can be considered to be a model for mini-columnar structure of the mammalian neocortex [19].

In order to find quasi-optimal network topologies, we have used (Sec. Results) a Monte Carlo evolution technique [20], assuming equal bidirectional coupling strengths. This evolving technique simulates the rewiring of a neuron network that maximizes or minimizes some cost function, in this case a cost function which produces quasi-optimal networks to transmit information.

Finally, we discuss how to extend these results to networks formed by elements that are non-chaotic (Sec. Results), and to non-autonomous networks, that are being perturbed by some time-dependent stimuli (Sec. Results).

## Results

### Upper bound for the Mutual Information Rate (MIR) in an Active Network

In a recent publication [10], we have argued that the mutual information rate (MIR) between two elements in an active chaotic network, namely, the amount of information per unit time that can be realized in one element, *k*, by measuring another element, *l*, regarded as *I_C_*, is given by the sum of the conditional Lyapunov exponents associated with the synchronization manifold (regarded as λ^∥^) minus the positive conditional Lyapunov exponents associated with the transversal manifold (regarded as λ^⊥^). So, *I_C_* = λ^∥^−λ^⊥^.

As shown in [11], if one has N = 2 coupled chaotic systems, which produce at most two positive Lyapunov exponents *λ*_1_, *λ*_2_ with λ_1_>λ_2_, then λ^∥^ = λ_1_ and λ^⊥^ = λ_2_. Denote the trajectory of the element *k* in the network by **x_k_**. For larger number of elements, *N*, the approaches proposed in [10] remain valid whenever the coordinate transformation **X_kl_**_∥_ = **x_k_**+**x_l_** (which defines the synchronization manifold) and **X_kl_**_⊥_ = **x_k_**−**x_l_** (which defines the transversal manifold) successfully separates the two systems *k* and *l* from the whole network. Such a situation arises in networks of chaotic maps of the interval connected by a diffusively (also known as electrically or linear) all-to-all topology, where every element is connected to all the other elements. These approaches were also shown to be approximately valid for chaotic networks of oscillators connected by a diffusively all-to-all topology. The purpose of the present work is to extend these approaches and ideas to active networks with arbitrary topologies.

Consider an active network formed by *N* equal elements, **x_i_** (*i* = 1,…,*N*), where every *D*-dimensional element has a different set of initial conditions, i.e., **x**_1_≠**x**_2_≠…≠**x** *N*. The network is described by
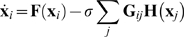
(1)where **G** *_ij_* is the *ij* element of the coupling matrix. Since we choose 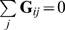 in order for a synchronization manifold to exist by the subspace **η** = **x**_1_ = **x**_2_ = **x**_3_ = … = **x**_*N*_, we can call this matrix the Laplacian matrix.

The way small perturbations propagate in the network [21] is described by the *i* (*i* = 1,…,*N*) variational equations of Eqs. (1), namely writing **x** *_i_* = *η*+*δ* **x** *_i_* and expanding Eq. (1) in *δ* **x** *_i_*,
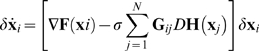
(2)obtained by linearly expanding Eq. (1).

Making **x** *_i_* = ξ, which can be easily numerically done by setting the elements with equal initial conditions and taking **H**(**x** *_j_*) = **x** *_j_*, Eq. (2) can be made block diagonal resulting in
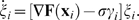
(3)where γ*_i_* are the eigenvalues (positive defined) of the Laplacian matrix ordered such that γ*_i_*_+1_≥γ*_i_*. Note that γ_1_ = 0.

Notice that the network dynamics is described by Eq. (1), which assumes that every element has different initial conditions and therefore different trajectories (except when the elements are completely synchronized). On the other hand, Eq. (3) that provides the conditional exponents considers that all the initial conditions are equal. While Eq. (2) provides the set of Lyapunov exponents of an attractor, Eq. (3) provides the Lyapunov exponents of the synchronization manifold and its transversal directions. Notice also that when dealing with linear dynamics, the Lyapunov exponents [obtained from Eq. (2)] are equal to the conditional exponents [obtained from Eq. (3)] independently on the initial conditions.

Then, the upper bound of the MIR that can be measured from an element **x_k_** by observing another element **x_l_**, i.e. the upper bound of the MIR in the communication channel *c^i^* ^−1^ is
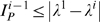
(4)with *i*∈(2,…,*N*), and λ*^i^* representing the sum of all the **positive** Lyapunov exponents of the equation for the mode ξ*_i_*, in Eq. (3). So, λ^1^ is the sum of the positive conditional exponents obtained from the separated variational equations, using the smallest eigenvalue associated with the exponential divergence between nearby trajectories around ξ, the synchronous state, and λ*^i^* (*i*>1) are the sum of the positive conditional exponents of one of the possible desynchronous oscillation modes. Each eigenvalue γ*_i_* produces a set of conditional exponents 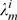, with *m* = 1,…,*D*.

Although Eq. (4) gives the upper bound for the amount of information between modes of oscillation, for some simple network geometries, as the ones studied here, we can relate the amount of information exchanged between two vibrational modes to the amount of information between two elements of the network, and therefore, Eq. (4) can be used to calculate an upper bound for the MIR exchanged between pairs of elements in the network. For larger and complex networks, this association is non-trivial, and we rely on the reasonable argument that a pair of elements in an active network cannot transmit more information than some of the *i*−1 values of 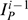.

The inequality in Eq. (4) can be interpreted in the following way. The right hand side of Eq. (4) calculates the amount of information that one could transmit if the whole network were completely synchronous with the state ξ, which is only true when complete synchronization takes place and when all the nodes have equal dynamics. Typically, we expect that the elements of the network will not be completely synchronous to ξ and in realistic networks, the nodes will not be equal. Thus, the amount of information provided by the right part of Eq. (4) overestimates the exact MIR which, due to desynchronization in the network, should be smaller than the calculated one.

Equation (5) allows one to calculate the MIR between oscillation modes of larger networks with arbitrary topology rescaling the MIR curve (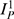 vs. σ) obtained from two coupled elements. Denoting σ^*^(*N* = 2) as the strength value for which the curve for λ^2^ reaches a relevant value, say, its maximum value, then the coupling strength for which this same maximum is reached for λ*^i^* in a network composed by *N* elements is given by
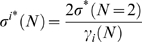
(5)where γ*_i_*(*N*) represents the *i*th largest eigenvalue of the *N*-elements network. If the network has an all-to-all topology, thus, σ^*^(*N* = 2) represents the strength value for which the curve of 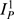 reaches a relevant value, and σ^*^(*N*) the strength value that this same value for 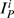 is reached.

Notice that symmetries in the connecting network topology leads to the presence of degenerate eigenvalues ( = equal eigenvalues) in the Laplacian matrix, which means that there are less independent channels of communication along which information flows. Calling *Q* the number of degenerate eigenvalues of the Laplacian matrix, Eq. (4) will provide *N*−*Q* different values.

As the coupling strength σ is varied, the quantities that measure information change correspondingly. For practical reasons, it is important that we can link the way these quantities (see Sec. Methods) change with the way the different types of synchronization show up in the network. In short, there are three main types of synchronization observed in our examples (see [11]): burst phase synchronization (BPS), when at least one pair of neurons are synchronous in the slow time-scale but desynchronous in the fast time-scale, phase synchronization (PS), when all pairs of neurons are phase synchronous, and complete synchronization (CS), when all pairs of neurons are completely synchronous. The coupling strength for which these synchronous phenomena appear are denoted by σ*_BPS_*, σ*_PS_*, and σ*_CS_* (with no superscript index).

Finally, there are a few more relevant coupling strengths, which characterize each communication channel. First, 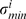, for which λ*^i^* equals the value of λ^1^, with *i*≥2. For 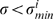, the communication channel *c^i^* ^−1^ (whose upper rate of information transmission depends on the two oscillation modes ξ_1_ and ξ*_i_*) behaves in a self-excitable way, i.e., λ^1^<λ*^i^*. For 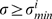, λ^1^≥λ*^i^*. Secondly, σ*^i^* ^*^ indicates the coupling strength at which 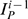 is maximal. Thirdly, 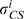 indicates the coupling strength for which the communication channel *c^i^* ^−1^ becomes “stable”, i.e., λ*^i^*<0. At σ = σ*^i^* ^*^ the self-excitable channel capacity of the channel *c^i^* ^−1^ is reached and at 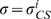, the non-self-excitable channel capacity is reached. Finally, σ*_C_* is the coupling for which the network capacity is reached, and then, when the KS-entropy of the network is maximal.

### The MIR in networks of coupled Hindmarsh-Rose neurons

We investigate how information is transmitted in self-excitable networks composed of *N* bidirectionally coupled Hindmarsh-Rose neurons [18]:
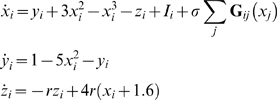
(6)The parameter *r* modulates the slow dynamics and is set equal to 0.005, such that each neuron is chaotic. The index *i*≠*j* assumes values within the set [1,…,*N*]. *S_k_* represents the subsystem formed by the variables (*x_k_*, *y_k_*, *z_k_*) and *S_l_* represents the subsystem formed by the variables (*x_l_*, *y_l_*, *z_l_*), where *k* = [1,…,*N*−1] and *l* = [*k*+1,…,*N*]. The Laplacian matrix is symmetric, so **G** *_ji_* = **G** *_ij_*, and *σ* **G** *_ji_* is the strength of the electrical coupling between the neurons, and we take for *I_i_* the value *I_i_* = 3.25.

In order to simulate the neuron network and to calculate the Lyapunov exponents through Eq. (2), we use the initial conditions *x* = −1.3078+η, *y* = −7.3218+η, and *z* = 3.3530+η, where η is an uniform random number within [0,0.02]. To calculate the conditional Lyapunov exponents, we use the equal initial conditions, *x* = −1.3078, *y* = −7.3218, and *z* = 3.3530.

#### All-to-all coupling

**Figure 1:**
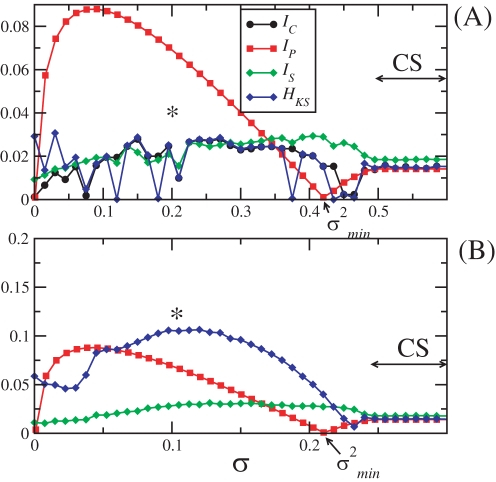
The quantities *I_C_* (black circles), *I_P_* (red squares), *I_S_* (green diamonds), and *H_KS_* (blue diamonds), for two (A) and four (B) coupled neurons, in an all-to-all topology. Notice that since there are only two different eigenvalues, there is only one channel of communication whose upper bound for the MIR is given by *I_P_* = |λ^1^−λ^2^|. Also, *I_S_* and *I_C_* represent the mutual information exchanged between any two pairs of elements in the system. In (A), σ^2*^ = 0.092, σ*_BPS_*≅0.2, 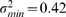, σ*_PS_* = 0.47, and σ*_CS_* = 0.5. In (B), σ^2*^ = 0.046, σ*_BPS_*≅0.1, 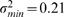, σ*_PS_* = 0.24, and σ*_CS_* = 0.25. CS indicates the coupling interval σ≥σ*_CS_* for which there exists complete synchronization.

Here, we analyze the case where *N* neurons are fully connected to every other neuron. The Laplacian matrix has *N* eigenvalues, γ_1_ = 0, and *N*−1 degenerate ones γ*_i_* = *N*, *i* = 2,…,*N*. Every pair of neurons exchange an equal amount of MIR. Although, there are *N*×(*N*−1)/2 pairs of neurons, there is actually only one independent channel of communication, i.e., a perturbation applied at some point of the network should be equally propagated to all other points in the network. In Fig. 1(A), we show the MIR, *I_C_*, calculated using the approaches in Refs. [10], [11], *I_P_*, calculated using the right hand-side of Eq. (4), and *I_S_*, calculated encoding the trajectory between pair of neurons, and the Kolmogorov-Sinai entropy, *H_KS_*, for a network composed by *N* = 2 neurons. In (B), we show these same quantities for a network formed by *N* = 4 neurons.

While for σ≅0 and σ≥σ*_CS_*, we have that *I_C_*≅*I_P_*≅*I_S_*, for σ≅σ^2*^ (when the self-excitable channel capacity is reached) it is clear that *I_P_* should be an upper bound for the MIR, since not only *I_P_*>*I_C_* but also *I_P_*>*I_S_*. Notice the good agreement between *I_C_* and *I_S_*, except for 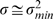, when *I_S_*>*H_KS_*, which violates Eq. (11).

The star symbol indicates the value of the coupling, σ*_BPS_*, for which burst phase synchronization (BPS) appears while the spikes are highly desynchronous. The appearance of BPS coincides with the moment where all the quantifiers for the MIR are large, and close to a coupling strength, σ*_C_*, for which the network capacity is reached (when *H_KS_* is maximal).

At this point, the network is sufficiently desynchronous to generate a large amount of entropy, which implies a large λ*^i^*, for *i*≥2. This is an ideal configuration for the maximization of the MIR. There exists phase synchrony in the subspace of the slow time-scale *z* variables (which is responsible for the bursting-spiking behavior), but there is no synchrony in the (*x*,*y*) subspace. This supports the binding hypothesis, a fundamental concept of neurobiology [19] which sustains that neural networks coding the same feature or object are functionally bounded. It also simultaneously supports the works of [22], which show that desynchronization seems to play an important role in the perception of objects as well. Whenever λ^2^ approaches zero, at σ = σ*_CS_*, there is a drastic reduction in the value of *H_KS_* as well as *I_P_*, since the network is in complete synchronization (CS), when all the variables of one neuron equals the variables of the other neurons.

Therefore, for coupling strengths larger than the one indicated by the star symbol, and smaller than the one where CS takes place, there is still one time-scale, the fast time-scale, which is out of synchrony.

For 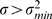, the only independent communication channel is of the non-self-excitable type. That means λ*^i^*≤λ^1^ (*i*≥2), and as the coupling strength increases, *H_KS_* decreases and *I_P_* increases.

Note that the curve for *I_P_* shown in Fig. 1(B) can be obtained by rescaling the curve shown in Fig. 1(A), applying Eq. (5).

#### Star coupling

We consider *N* = 4. There is a central neuron, denoted by *S*_1_, bidirectionally connected to the other three (*S_k_*, *k* = 2,3,4), but none of the others are connected among themselves. The eigenvalues of the Laplacian matrix are *γ*_1_ = 0, *γ*_2,3_ = 1, *γ*_4_ = *N*.

To treat general types of networks, it is useful to define two quantities related to the excitability of the communication channels. The here called non-self-excitable (NSE) robustness parameter of the channel *c^i^* ^−1^ (*i*≥2) as 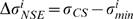 and the self-excitable (SE) robustness parameter for the communication channel *c^i^* ^−1^ as 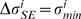 (*i*≥2). It is also useful to define a quantity that measures the distance between the eigenvalues, the normalized spectral distance (NED) between the two eigenvalues.

Having a large NED between the *i*th largest and the first largest eigenvalues (*γ_i_*−*γ*_2_)/*N*, results in a non-self-excitable channel, *c^i^* ^−1^, with a large NSE robustness parameter that implies that the channel preserves its NSE character under large alterations of the coupling strength. On the other hand, having a large NED between the largest and the *i*th largest eigenvalues (γ*_N_*−γ*_i_*)/*N*, results in a self-excitable channel, *c^i^* ^−1^, with a large self-excitable robustness parameter that implies that the channel preserves its SE character under large alterations of the coupling strength.

So, for the star topology network, not only the NED between γ*_N_* and γ*_N_*_−1_ is large but also between γ*_N_* and γ*_N_*_−2_, and therefore, 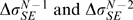 are large. This provides a network whose channels *c* ^1^ and *c* ^2^ have a large MIR for a large coupling strength alteration. Note that if γ*_N_*_−1_ is far away from γ*_N_* that implies that γ*_N_*_−2_ is also far away from γ*_N_*. Thus, a reasonable spectral distance between γ*_N_*_−1_ and γ*_N_* is a “biological requirement” for the proper function of the network, since even for larger coupling strengths there will be at least one oscillation mode which is desynchronous, a configuration that enables perturbation (meaning external stimuli) to be propagated within the network [23].

The largest eigenvalue is related to an oscillation mode where all the outer neurons are in synchrony with each other but desynchronous with the central neuron. So, here it is clear the association between |λ^1^−λ^4^| and the MIR between the central neuron with an outer neuron, since λ^1^ represents the amount of information of the synchronous trajectories among all the neurons, while λ^4^ is the amount of information of the desynchronous trajectories between the central neuron and any outer neuron. The other eigenvalues (γ_2_,γ_3_) represent directions transverse to the synchronization manifold in which the outer neurons become desynchronous with the central neuron in waves wrapping commensurately around the central neuron [21]. Thus, λ^2^ and λ^3^ are related to the error in the transmission between two outer neurons, *k* and *l*, with *k*,*l*≠1. Notice that this network topology has two independent channels of communication.

**Figure 2:**
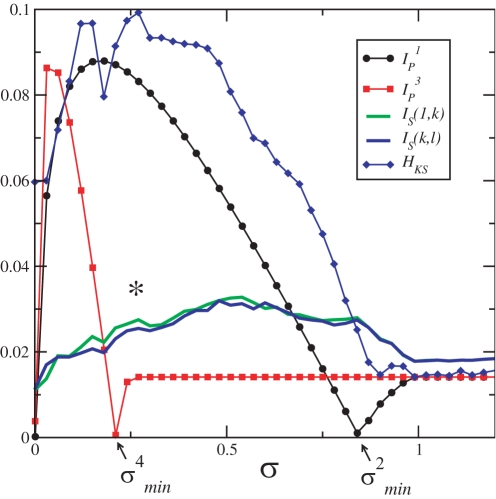
MIR between the central neuron and an outer one (black circles), 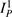, (resp. *I_S_* (1, *k*), in green line), and between two outer ones (red squares), 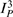, (resp. *I_S_* (*k*, *l*), in blue line). Blue diamonds represents the KS-entropy. Other quantities are σ^4*^ = 0.181, σ^2*^ = 0.044, 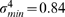, 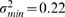, 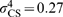, σ*_BPS_* = 0.265, σ*_PS_* = 0.92, and σ*_CS_* = 1.0. The star indicates the parameter for which BPS first appears.

Note that the MIR between *S*_1_ and an outer neuron (upper bound represented by 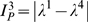 and *I_S_* represented by *I_S_* (1, *k*), in Fig. (2) is larger (smaller) than the MIR between two outer neurons (upper bound represented by 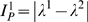 and *I_S_* is represented by *I_S_* (*k*, *l*), in Fig. (2), for small coupling (for when the channel *c* ^3^ is self-excitable, and 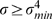). Similar to what happens to nearest-neighbour networks, the self-excitable and the non-self-excitable channel capacities of the channel associated with the transmission of information between closer elements (the channel *c* ^3^) are achieved for a smaller value of the coupling strength than the one necessary to make the channels associated with the transmission of information between more distant elements (the channel *c* ^1^) to achieve its two channel capacities. That property permits this network, for 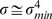, to transmits simultaneously reliable information using the channel *c* ^3^ and with a higher rate using the channel *c* ^1^.

Notice, in Fig. 2, that 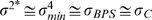. So, when the channel capacity of the channel *c* ^1^ is reached, also *H_KS_* of the network is maximal, and the network operates with its capacity.

Another point that we want to emphasize in this network is that while a large NED between γ*_N_* and γ*_N_*_−1_ provides a network whose channel *c* ^1^ is self-excitable and can transmit information at a large rate for a large coupling strength interval, a large NED between γ_3_ and γ_2_ leads to a non-self-excitable channel *c* ^3^ even for small values of the coupling amplitudes, and it remains non-self-excitable for a large variation of the coupling strength. Thus, while a large NED between the second and the first largest eigenvalues leads to a network whose channels are predominantly of the self-excitable types, a large NED between the second largest and the third largest eigenvalues provide a network whose communication channels are predominantly of the non-self-excitable types.

### Eigenvalues conditions

Finding network topologies and coupling strengths in order to have a network that operates in a desired fashion is not a trivial task (see Sec. Methods). An ideal way to proceed would be to evolve the network topology in order to achieve some desired behaviour. In this paper, we are interested in maximizing simultaneously *I_P_*, the KS-entropy, and the average 〈*I_P_*〉, for a large range of the coupling strength, characteristics of a *quasi-optimal network*. However, evolving a network in order to find a quasi-optimal one would require the calculation of the MIR in every communication channel and *H_KS_* for every evolution step. For a typical evolution, which requires 10^6^ evolution steps, such an approach is impractical.

Based on our previous discussions, however, a quasi-optimal network topology can be realized by only selecting an appropriate set of eigenvalues which have some specific NED. Evolving a network by the method of Sec. Methods using a cost function which is a function of only the eigenvalues of the Laplacian matrix is a practical and physible task.

The present section is dedicated to describe the derivation of this cost function.

**Figure 3:**
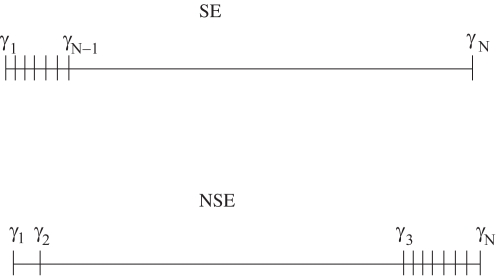
Representation of the eigenvalues sets that produce quasi-optimal self-excitable (SE) and non-self-excitable active networks (NSE).

We can think of two most relevant sets of eigenvalues which create quasi-optimal networks, and they are represented in Fig. 3. Either it is desired eigenvalues that produce a network predominantly self-excitable [SE, in Fig. 3] or predominantly non-self-excitable [NSE, in Fig. 3].

In a network whose communication channels are predominantly self-excitable, it is required that the NED (γ*_N_*−γ*_N_*_−1_)/*N* is maximal and (γ*_N_*_−1_)/*N* minimal. Therefore, we want a network for which the cost function
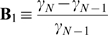
(7)is maximal.

A network whose eigenvalues maximize **B**_1_ has self-excitable channels for a large variation of the coupling strength. As a consequence, 〈*I_P_*〉 as well as *H_KS_* is large for 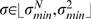.

In a network whose communication channels are predominantly non-self-excitable, it is required that the NED (γ_3_−γ_2_)/*N* is maximal and (γ_2_)/*N* minimal. Therefore, we want a network for which the cost function
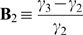
(8)is maximal.

A network whose eigenvalues maximize the condition in Eq. (8) have non-self-excitable channels for a large variation of the coupling strength. As a consequence, 〈*I_P_*〉 is large for 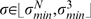, which is a small coupling range, but since there is still one oscillation mode that is unstable (the mode ξ_2_), *H_KS_* is still large for a large range of the coupling strength 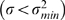. Most of the channels will transmit information in a reliable way, since the error in the transmission, provided by λ*^i^* (*i*≥2), of most of the channels will be zero, once λ*^i^*<0.

Since degenerate eigenvalues produce networks with less vibrational modes and therefore less independent channels of communication, we assume in the following the absence of such degenerate eigenvalues. In addition, we assume that there is a finite distance between eigenvalues so that the network becomes robust under rewiring, and therefore, perturbing **G** *_ij_* will not easily create degenerate eigenvalues.

A network that is completely synchronous and has no unstable modes does not provide an appropriate environment for the transmission of information about an external stimulus, because they prevent the propagation of perturbations. Networks that can be easily completely synchronized (for small coupling strengths) requires the minimization of γ*_N_*−γ_2_, or in terms of the eigenratio, the minimization of γ*_N_*/γ_2_. We are not interested in such a case. To construct network topologies that are good for complete synchronization, see Refs. [21], [24], [25], [26].

### Quasi-optimal topologies for information transmission

Before explaining how we obtain quasi-optimal network topologies for information transmission, it is important to discuss the type of topology expected to be found by maximizing either **B_1_**, in Eq. (7) or **B_2_**, in Eq. (8). Notice that Laplacians whose eigenvalues maximize **B_1_** are a perturbed version of the star topology, and the ones that maximize **B**_2_ are a perturbed version of the all-to-all topology. In addition, in order to have a network that presents many independent modes of oscillations it is required that the Laplacian matrix presents as much as possible, a large number of non-degenerate eigenvalues. That can be arranged by rewiring (perturbing) networks possessing either the star or the nearest-neighbour topology, breaking the symmetry.

In order to calculate a Laplacian from a quasi-optimal network, we propose an approach described in Sec. Methods, based on the reconstruction of the network by evolving techniques, simulating the process responsible for the growing or rewiring of real biological networks, a process which tries to maximize or minimize some cost function.

**Figure 4:**
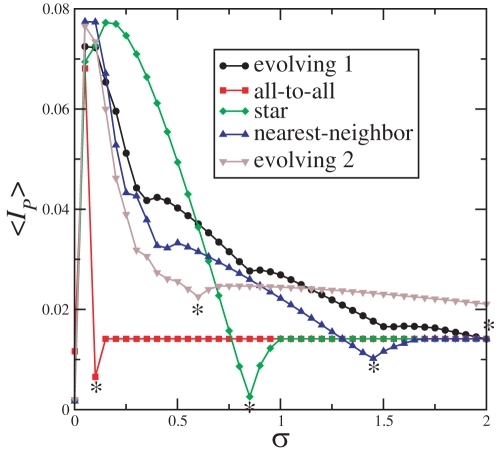
The average value of the upper bound MIR, 〈*I_P_*〉 [as defined in Eq. (10)] for active networks composed of 8 elements using one of the many topologies obtained by evolving the network maximizing B_1_ (circles), all-to-all topology (squares), star topology (diamonds), nearest-neighbor (upper triangle), and maximizing B_2_ (down triangle). The values of 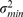 indicated by the starts are 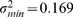 (evolving 1), 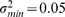 (all-to-all), 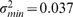 (star), 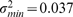 (nearest-neighbor), and 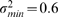 (evolving 2). The evolving 1 network has a Laplacian with relevant eigenvalues γ_7_ = 3.0000, γ_8_ = 6.1004, which produces a cost function equal to B_1_ = 1.033. The evolving 2 network has a Laplacian with relevant eigenvalues γ_2_ = 0.2243 and γ_3_ = 1.4107, which produces a cost function equal to B_2_ = 5.2893.

In order to better understand how a network evolves (grows) in accordance with the maximization of the cost functions in Eqs. (7) and (8), we first find the network configurations with a small number of elements. To be specific, we choose *N* = 8 elements. To show that indeed the calculated network topologies produce active networks that operate as desired, we calculate the average upper bound value of the MIR [Eq. (10)] for neural networks described by Eqs. (6) with the topology obtained by the evolution technique, and compare with other network topologies. Figure 4 shows 〈*I_P_*〉, the average channel capacity, calculated for networks composed of 8 elements, using one of the many topologies obtained by evolving the network maximizing **B_1_** (circles, denoted in Fig. by “evolving 1”), all-to-all topology (squares), star topology (diamonds), nearest-neighbor (upper triangle), and maximizing **B**_2_ (down triangle, denoted in Fig. by “evolving 2”). The star points to the value of 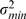, when *c* ^1^, the most unstable communication channel (a self-excitable channel), becomes non-self-excitable.

As desired the evolving network 1 has a large upper bound for the MIR (as measured by 〈*I_P_*〉) for a large range of the coupling strength, since the network has predominantly self-excitable channels. The channel *c* ^1^ has a large robustness parameter 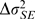, i.e., it is a self-excitable channel for 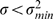, where 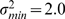. In contrast to the other topologies, in the star, nearest-neighbour, and all-to-all topologies, 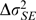 is smaller and 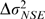 is larger. Even though most of the channels in the evolving 2 topology are of the non-self-excitable type, 〈*I_P_*〉 remains large even for higher values of the coupling strength. That is due to the channel *c* ^1^ which turns into a self-excitable channel only for σ>2.

**Figure 5:**
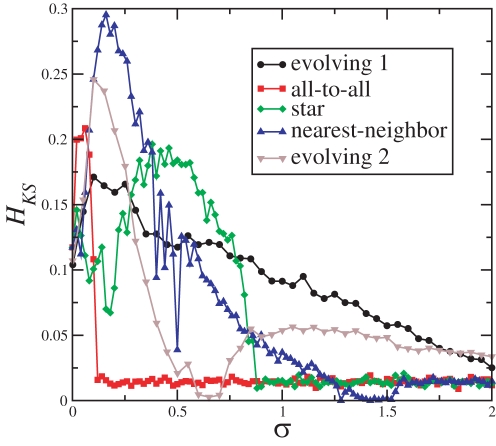
KS-entropy for the same active networks of Fig. 4 composed of 8 elements.

The KS-entropies of the 5 active networks whose 〈*I_P_*〉 are shown in Fig. 4 are shown in Fig. 5. Typically, the network capacities are reached for roughly the same coupling strength for which the maximum of 〈*I_P_*〉, is reached. In between the coupling strength for which the network capacities and the maximal of 〈*I_P_*〉 are reached, λ^3^ becomes negative. At this point, also BPS appears in the slow time-scale, suggesting that this phenomena is the behavioral signature of a network that is able to transmit not only large amounts of information between pairs of elements (high MIR) but also overall within the network (high *H_KS_*).

Note however, that since the evolving networks have a small number of elements, the cost function cannot reach higher values and therefore, the networks are not as quasi-optimal as they can be. For that reason, we proceed now to evolve larger networks, with *N* = 32.

**Figure 6:**
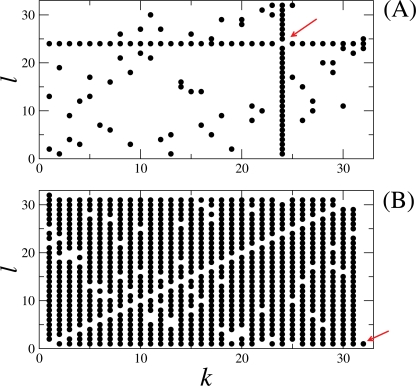
A point in this figure in the coordinate *k*×*l* means that the elements *S_k_* and *S_l_* are connected with equal couplings in a bidirectional fashion. In (A), a 32 elements network, constructed by maximizing the cost function B_1_ in Eq. (7) and in (B), 32 elements network, constructed by maximizing the cost function B_2_ in Eq. (8). In (A), the network has the topology of a perturbed star, a hub of neurons connected to all the other neurons, where each outer neuron is sparsely connected to other neurons. The arrow points to the hub. In (B),the network has the topology of a perturbed all-to-all network, where elements are almost all-to-all connected. Note that there is one element, the neuron *S*_32_, which is only connected to one neuron, the *S*_1_. This isolated neuron is responsible to produce the large spectral gap between the eigenvalues γ_3_ and γ_2_. In (A), the relevant eigenvalues are γ_31_ = 4.97272, γ_32_ = 32, which produce a cost function equal to B_1_ = 5.43478. In (B), the relevant eigenvalues are γ_2_ = 0.99761, γ_3_ = 27.09788, which produce a cost function equal to B_2_ = 26.1628.

Maximization of the cost function **B_1_** leads to the network connectivity shown in Fig. 6(A) and maximization of the cost function **B**_2_ leads to the network connectivity shown in Fig. 6(B). In (A), the network has the topology of a perturbed star, a neuron connected to all the other outer neurons, thus a hub, and each outer neuron is sparsely connected to other outer neurons. The arrow points to the hub. In (B),the network has the topology of a perturbed all-to-all network, where elements are almost all-to-all connected. Note that there is one element, the neuron *S*_32_, which is only connected to one neuron, the *S*_1_. This isolated neuron is responsible to produce the large spectral gap between the eigenvalues γ_3_ and γ_2_.

**Figure 7:**
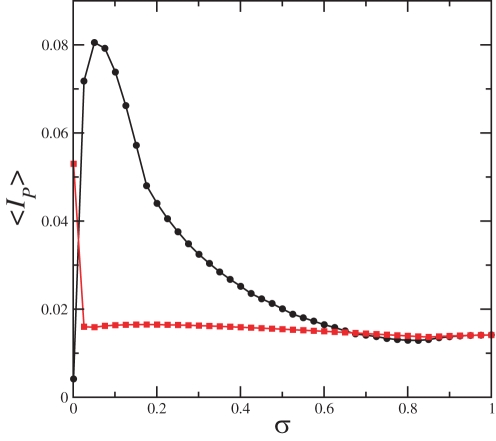
〈*I_P_*〉 for the networks shown in Fig. 6(A–B) by circles and squares, respectively.

〈*I_P_*〉 for the network topology represented in Fig. 6(A) is shown in Fig. 7 as circles, and 〈*I_P_*〉 for the network topology represented in Fig. 6(B) is shown in Fig. 7 as squares. We see that the star topology, whose connectivity is represented in 6(A), has larger 〈*I_P_*〉 for a larger coupling strength than the topology whose connectivity is represented in 6(B). Other relevant parameters of the network whose topology is represented in 6(A) are 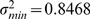, 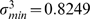, 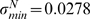, σ*_CS_* = 0.9762 and for the topology represented in 6(B) are 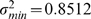, 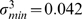, 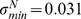, and σ*_CS_* = 0.9761.

It is worth to comment that the neocortex is being simulated in the Blue Brain project, by roughly creating a large network composed of many small networks possessing the star topology. By doing that, one tries to recreate the way minicolumnar structures [19] are connected to minicolumnar structures of the neocortex [27]. Each minicolumn can be idealized as formed by a pyramidal neuron (the hub) connected to its interneurons, the outer neurons in the star topology, which are responsible for the connections among this minicolumn (small network) to others minicolumn. So, the used topology to simulate minicolumns is an good topology in what concerns the transmission of information.

### Active networks formed by non-chaotic elements

The purpose of the present work is to describe how information is transmitted via an active media, a network formed by dynamical systems. There are three possible asymptotic stable behaviours for an autonomous dynamical system: chaotic, periodic, or quasi-periodic. A quasi-periodic behaviour can be usually replaced by either a chaotic or a periodic one, by an arbitrary perturbation. For that reason, we neglect such a state and focus the attention on active channels that are either chaotic or periodic.

Equation (4) is defined for positive exponents. However, such an equation can also be used to calculate an upper bound for the rate of mutual information in systems that also possess negative Lyapunov exponents. Consider first a one-dimensional contracting system being perturbed by a random stimulus. Further consider that the stimulus changes the intrinsic dynamics of this system. This mimics the process under which an active element adapts to the presence of a stimulus.

Suppose the stimulus, θ*_n_*, can be described by a discrete binary random source with equal probabilities of generating ‘0’ or ‘1’. Whenever θ*_n_* = 0, the system presents the dynamics *x_n_*_+1_ = *x_n_*/2, otherwise *x_n_*_+1_ = (1+*x_n_*)/2. It is easy to see that the only Lyapunov exponent of this mapping, λ_1_, which is equal to the conditional exponent, λ^1^, is negative. Negative exponents do not contribute to the production of information. From Eq. (4) one would arrive at *I_P_* = 0. However, all the information about the stimulus is contained in the trajectory. If one measures the trajectory *x_n_*, one knows exactly what the stimulus was, either a ‘0’ or a ‘1’. The amount of information contained in the stimulus is log(2) per iteration which equals the absolute value of the Lyapunov exponent, |λ_1_|. In fact, it is easy to show that *I_C_* = *I_P_* = |λ^1^| = |λ_1_| = log(2), or if we use the interpretation of [28], *I_C_* = *I_P_* = λ, where λ = |λ_1_| is the positive Lyapunov exponent of the time-inverse chaotic trajectory, *x_n_*_+*m*_, *x_n_*_+*m*−1_, …, *x*_0_, which equals the rate of information production of the random source. So, in this type of active communication channel, one would consider in Eq. (4) the positive Lyapunov exponents of the time-inverse trajectory, or the absolute value for the negative Lyapunov exponent.

Another example was given in [11]. In this reference we have shown that a chaotic stimulus perturbing an active system with a space contracting dynamics (a negative Lyapunov exponent) might produce a fractal set. We assume that one wants to obtain information about the stimulus by observing the fractal set. The rate of information retrieved about the stimulus on this fractal set equals the rate of information produced by the fractal set. This amount is given by D_1_|λ|, where *D*_1_ is the information dimension of the fractal set and |λ| the absolute value of the negative Lyapunov exponent. In fact, D_1_|λ| is also the rate of information produced by the stimulus. So, if an active system has a space contracting dynamics, the channel capacity equals the rate of information produced by the stimulus. In other words, the amount of information that the system allows to be transmitted equals the amount of information produced by the chaotic stimulus.

### The role of a time-dependent stimulus in an active network

The most general way of modelling the action of an arbitrary stimulus perturbing an active network is by stimulating it using uncorrelated white noise. Let us assume that we have a large network with all the channels operating in non-self-excitable fashion. We also assume that all the transversal eigenmodes of oscillations except one are stable, and therefore do not suffer the influence of the noise. Let us also assume that the noise is acting only on one structurally stable ( = far from bifurcation points) element, *S_k_*. To calculate the upper bound of the MIR between the element *S_k_* and another element *S_l_* in the network, we assume that the action of the noise does not alter the value of λ^1^. Then, the noise on the element *S_k_* is propagated along the vibrational mode associated with the one unstable transversal direction, whose conditional exponent is λ^2^. As a consequence, the action of the noise might only increase λ^2^, while not affecting the negativeness of all the other exponents (λ*^m^*, *m*>2), associated with stable transversal modes of oscillation. That means that the channels responsible for transmitting large amounts of information (associated with λ*^m^*, with *m* large) will not be affected. So, for such types of noises, Eq. (4) of the autonomous network is an upper bound for the non-autonomous network.

Consider now a situation where the noise acts equally on all the elements of an active network. The mapping
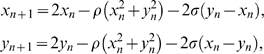
(9)was proposed as a way to understand such a case. In this mapping, we consider ρ≥0 and *x_n_*, *y_n_*∈[0,1], which can be accomplished by applying the *mod*(1) operation.

Note that the term 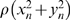 that enters equally in all the maps has statistical properties of an uniformly distributed random noise. Calculating *I_P_* for ρ = 0 (the noise-free map) we arrive at *I_P_*≅2σ, for small σ, while the true MIR *I_C_*≅2(σ−ρ). These results are confirmed by exact numerical calculation of the Lyapunov exponents of Eq. (9) as well as the calculation of the conditional exponents of the variational equations. So, this example suggests that Eq. (4) calculated for an autonomous non-perturbed network gives the upper bound for the mutual information rate in a non-autonomous network.

## Discussion

We have shown how to relate in an active network the rate of information that can be transmitted from one point to another, regarded as mutual information rate (MIR), the synchronization level among elements, and the connecting topology of the network. By active network, we mean a network formed by elements that have some intrinsic dynamics and can be described by classical dynamical systems, such as chaotic oscillators, neurons, phase oscillators, and so on.

Our main concern is to suggest how to construct a quasi-optimal network. A network that simultaneously transmits information at a large rate, is robust under couplings alterations, and further, it possesses a large number of independent channels of communication, pathways along which information travels.

We find that there is not the best topology but many that can be classified in two classes. Self-excitable [maximizing Eq. (7)] or non-self-excitable [maximizing Eq. (8)] (see definition of self-excitability in Sec. Methods). Self-excitable networks have communication channels that transmit information in a higher rate for a large range of the coupling strength. Most of the oscillation modes in these networks are unstable, and therefore, information is mainly propagated in a desynchronous environment. Non-self-excitable networks have communication channels that transmit information in a higher rate for a small range of the coupling strength, however, they have channels that transmit reliable information in a moderate rate for large range of coupling strengths. Most of the oscillation modes in these networks are stable, and therefore, information is mainly propagated in a synchronous environment, a highly reliable environment for information transmission.

One of the main results of our work, the Eq. (4), which relates synchronization, topology and information in active networks, can only be used in networks composed of nodes that have equal dynamics. We have reasons to believe that if the nodes have non equal dynamics, Eq. (4) provides an upper bound for the value of the mutual information rate that modes in the network exchange. That was shown in Ref. [11] for two linear coupled maps. Another reason is given in the following. When the nodes are not completely synchronous, networks of nodes with equal dynamics but randomly coupled (as the networks in [12]) in Ref. [12]), are good models of networks with nodes that have different dynamics. We have found that these random networks with nodes electrically connected usually become more non-self-excitable than the networks with nodes being connected with equal bidirectional couplings. As a consequence, both the network capacity and the channel capacities become smaller. It remains still to be verified if that is so for networks whose nodes are connected with chemical synapses. As shown in Ref. [12], chemical couplings make the network to become highly excited. As a consequence, it might be that as the nodes are made non-equal, the network gains a self-excitable character, resulting in an increase of the information capacities. In such a case, Eq. (4) would provide a lower bound for the mutual information rate of networks with nodes that have non equal dynamics.

If brain-networks somehow grow in order to maximize the amount of information transmission, simultaneously remaining very robust under coupling alterations, the minimal topology that small neural networks must have should be similar to the one in Fig. 6(A), i.e., a network with a star topology, presenting a central element, a hub, very well connected to other outer elements, which are sparsely connected.

## Methods

### Self-excitability

In Ref. [11] self-excitability was defined in the following way. An active network formed by *N* elements, is said to be self-excitable if *H_KS_* (*N*, *σ*)>*H_KS_* (*N*, *σ* = 0), which means that the KS-entropy of the network increases as the coupling strength is increased. Thus, for non self-excitable systems, an increase in the coupling strength among the elements forming the network leads to a decrease in the KS-entropy of the network.

Here, we adopt also a more flexible definition, in terms of the properties of each communication channel. We define that a communication channel *c^i^* behaves in a self-excitable fashion if λ*^i^*>λ^1^. It behaves in a non-self-excitable fashion if λ*^i^*≤λ^1^.

### Mutual Information Rate (MIR), channel capacity, and network capacity

In this work, the rate with which information is exchanged between two elements of the network is calculated by different ways. Using the approaches of Refs. [10], [11], we can have an estimate of the real value of the MIR, and we refer to this estimate as *I_C_*. Whenever we use Eq. (4) to calculate the upper bound for the MIR, we will refer to it as *I_P_*. Finally, whenever we calculate the MIR through the symbolic encoding of the trajectory, we refer to it as *I_S_*.

We define the *channel capacity* of a communication channel formed by two oscillation modes depending on whether the channel behaves in a self-excitable fashion or not. So, for the studied network, every communication channel possess two channel capacities, the self-excitable capacity and the non-self-excitable one. A channel *c^i^* operates with its self-excitable capacity when 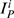 is maximal, what happens at the parameter σ^(*i*+1)*^. It operates with its non-self-excitable capacity when λ*^i^* ^+1^ = 0.

We also define the channel capacity in an average sense. In that case, the averaged channel capacity is given by the maximal value of the average value
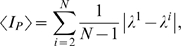
(10)


The *network capacity* of a network composed of *N* elements, *C_N_*(*N*), is defined to be the maximum value of the Kolmogorov-Sinai (KS) entropy, *H_KS_*, of the network. For chaotic networks, the KS-entropy, as shown by Pesin [29], is the sum of all the positive Lyapunov exponents. Notice that if *I* denotes the MIR then
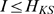
(11)


As shown in Ref. [11] and from the many examples treated here, *C_N_*(*N*)∝*N*, and so, the network capacity grows linearly with the number of elements in an active network.

### Understanding Eq. (4): Positiveness of the MIR for self-excitable channels in the (non-linear) HR network

To show that indeed 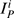 should be positive in case of a self-excitable channel in the HR network, one can imagine that in Eq. (1) the coupling strength is arbitrarily small and that *N* = 2. At this situation, the Lyapunov exponent spectra obtained from Eq. (2) are a first-order perturbative version of the conditional exponents, and they appear organized by their strengths. One arrives at λ_1_≅λ^2^ and λ_2_≅λ^1^, which means that the largest Lyapunov exponent equals the transversal conditional exponent and the second largest Lyapunov exponent equals the conditional exponent associated with the synchronous manifold. Using similar arguments to the ones in Refs. [10], [11], [30], we have that the MIR is given by the largest Lyapunov exponent minus the second largest, and therefore, *I_C_* = λ_1_−λ_2_, which can be put in terms of conditional exponents as *I_P_*≤λ^2^−λ^1^, or as represented in Eq. (4), *I_P_*≤|*λ* ^1^−*λ* ^2^|.

### Understanding Eq. (4): The inequality in Eq. (4)

To explain the reason of the inequality in Eq. (4), consider the following two coupled maps:
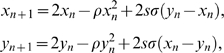
(12)with *s* = 1 and *x_n_*, *y_n_*∈[0,1]. For this mapping, the MIR can be written in terms of the Lyapunov Exponents [11], [31]. For two coupled systems, the MIR can be exactly calculated by *I_C_* = λ_1_−λ_2_, since λ^∥^ = λ_1_ and λ^⊥^ = λ_2_, assuming that both λ_1_ and λ_2_ are positive. Calculating the conditional exponents numerically, we can show that *I_P_*≥*I_C_*, and thus *I_P_* is an upper bound for the MIR. For more details on this inequality, see [12]

### Evolutionary construction of a network

In our simulations, we have evolved networks of equal bidirectional couplings [32]. That means that the Laplacian in Eq. (1) is a symmetric matrix of dimension *N* with integer entries {0,1} for the off diagonal elements, and the diagonal elements equal to 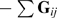, with *i*≠*j*.

Finding the network topologies which maximize **B** in Eq. (7) is impractical even for moderately large *N*. Figuring out by “brute force” which Laplacian produces the desired eigenvalue spectra would require the inspection of a number of 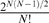 configurations. To overcome this difficulty, Ref. [20] proposed an evolutionary procedure in order to reconstruct the network in order to maximize some cost function. Their procedure has two main steps regarded as *mutation* and *selection*. The mutation steps correspond to a random modification of the pattern of connections. The selection steps consist in accepting or rejecting the mutated network, in accordance with the criterion of maximization of the cost function **B**, in Eq. (7).

We consider a random initial network configuration, with *N* elements, which produce an initial Laplacian **G**_0_, whose eigenvalues produce a value **B**_0_ for the cost function. We take at random one element of this network and delete all links connected to it. In the following, we choose randomly a new degree *k* to this element and connect this element (in a bidirectional way) to *k* other elements randomly chosen. This procedure generates a new network that possesses the Laplacian **G**′, whose eigenvalues produce a value **B**′. To decide if this mutation is accepted or not, we calculate Δ*ε* = **B**′−**B**_0_. If Δε>0, the new network whose Laplacian is **G**′ is accepted. If, on the other hand, Δε<0, we still accept the new mutation, but with a probability *p*(Δε) = exp(−Δε/*T*). If a mutation is accepted then the network whose Laplacian is **G**_0_ is replaced by the network whose Laplacian is **G**′.

The parameter *T* is a kind of “temperature” which controls the level of noise responsible for the mutations. It controls whether the evolution process converges or not. Usually, for high temperatures one expects the evolution never to converge, since new mutations that maximizes **B** are often not accepted. In our simulations, we have used *T*≅0.0005.

These steps are applied iteratively up to the point when |Δε| = 0 for about 10,000 steps, being that we consider an evolution time of the order of 1,000,000 steps. That means that the evolution process has converged after the elapse of some time to an equilibrium state. If for more than one network topology |Δε| = 0 for about 10,000 steps, we choose the network that has the larger **B** value.

This constraint avoids the task of finding the best network topology. However, we consider that a reasonably low number of mutations would recreate what usually happens in real networks.
